# Assessment of Outcomes by Intention-to-Treat Comparison for Locally Advanced Pancreatic Cancer: A Population-Derived Cohort Study

**DOI:** 10.1245/s10434-024-16291-9

**Published:** 2024-10-04

**Authors:** Patrik Larsson, Oskar Swartling, Diana Cheraghi, Ajnon Khawaja, Kjetil Soreide, Ernesto Sparrelid, Poya Ghorbani

**Affiliations:** Division of Surgery and Oncology, Department of Clinical Sciences, Interventions and Technology, Karolinska Institutet, Karolinska University Hospital, Stockholm, Sweden

**Keywords:** Pancreatic cancer, Locally advanced pancreatic cancer, Survival, Mortality, Chemotherapy, Surgery

## Abstract

**Background:**

The overall treatment response among patients with locally advanced pancreatic cancer (LAPC) is poorly understood as most studies report solely on resected patients. We aimed to investigate the outcomes in patients with LAPC as an intention-to-treat-analysis from the time of diagnosis from a complete source population.

**Patients and Methods:**

An observational cohort study in a population-defined region within a universal healthcare system. All consecutive patients discussed at multi-disciplinary tumour board (MDT), aged ≥ 18 years and diagnosed with LAPC were included. Exposure was set as recommended treatment by MDT (i.e. upfront surgery, neoadjuvant therapy, palliative treatment or best supportive care). Outcome measures were overall survival analysed by Kaplan–Meier survival estimates and multivariable analyses using logistic regression for odds ratios (OR) and Cox proportional hazard analysis for hazard ratios (HR).

**Results:**

In total, 8803 MDT events (6055 unique patients) with pancreatic disease were held during the study period. Some 1436 (24%) had pancreatic cancer, of which 162 (11%) had LAPC and 134 met the population-defined criteria. In overall survival analyses, the patients who were recommended neoadjuvant therapy (± surgery) demonstrated no significant difference to palliative chemotherapy (median 11.0 months vs. 11.8 months; *p* = 0.226). In multivariable analysis, adjusted OR for overall survival comparing the treatment groups was 0.27 (95% CI 0.02–3.29, *p* = 0.306) and Cox proportional HR 0.96 (95% CI 0.58–1.59, *p* = 0.865).

**Conclusions:**

In patients with LAPC, survival was not statistically different between those recommended for attempt at neoadjuvant (± surgery) compared with those recommended palliative chemotherapy. The findings suggest that conversion/downstaging chemotherapy is successful in only a select few.

**Supplementary Information:**

The online version contains supplementary material available at 10.1245/s10434-024-16291-9.

Pancreatic cancer has an overall poor prognosis, with few patients presenting at time of diagnosis in a disease stage that allows for surgical treatment.^1^ While up to half of all patients present with distant metastasis, an estimated 15% are amenable to upfront surgery, while further 10–15% may present as borderline and a further 20–30% as locally advanced pancreatic cancer (LAPC).^2^ The latter group is in principle not resectable unless stable disease or cancer response can be demonstrated after chemotherapy. Hence, the general recommendation is to start with neoadjuvant (or, alternatively called conversion or induction therapy) chemotherapy to select those who are responders (or have stable disease) and demonstrate no progress on chemotherapy before surgical exploration.^2–4^

Previous studies have reported on use of different neoadjuvant chemotherapy regimens, including mFOLFIRINOX or Gemcitabine-based combinations^5–8^ with associated differences in outcome and resection rates. Also, institutional series report on high resection rates, favourable survival after completion of all sequences, and comparison with treatment groups that may have variable arguments for use in back-to-back comparison. Some studies are subject to bias in inclusion criteria or report only on those few patients who tolerated all recommended treatments and had a favourable outcome, i.e. there may be a risk of publication bias.^9–11^ Therefore, studies using a selected approach to locally advanced pancreatic cancer analysis may not reflect the real-world situation of “all comers” with locally advanced pancreatic cancer and hence inflate the expectations and result presented to patients and surgeons alike. Of note, a recent nationwide Dutch study found that only 142 patients underwent surgery for locally advanced pancreatic cancer over a 7-year period covering 16 centres,^12^ highlighting the relative rarity of resections for locally advanced pancreatic cancer in a population-derived setting. In the Dutch study, the patients reported were the ones treated by surgery, and hence not considering patients with locally advanced pancreatic cancer who were offered other routes of treatment (i.e. palliative or best supportive care) based on presentation at the multidisciplinary treatment conference (MDT). As a result, solitary presentation of only resected patients does not tell how this group compares to those not offered surgery or a trial of neoadjuvant/induction therapy.

There is also a need to consider the perspectives on relevant outcome measures used to evaluate neoadjuvant treatment in locally advanced pancreatic cancer. For surgeons, tumour resection rate may be regarded as a proxy for response to neoadjuvant treatment, and resection is then often held in regards to patients not being resected to determine effectiveness.^8,13,14^ Comparing patients who undergo resection to those who do not may, however, reflect only a small part of the whole picture of the disease. Studying the outcome from an intention-to-treat approach could give additional insights on what the outcome may be for patients presenting with locally advanced pancreatic cancer. One of many challenges in previous studies has been to study the outcome for patients with locally advanced pancreatic cancer with a high external validity, i.e. does the selected patient group represent the situation reported at other centres or does it rather reflect merely a small, selected part of the entire group of patients with locally advanced pancreatic cancer. For a more complete overview of outcomes in locally advanced pancreatic cancer, this mandates a study cohort that reflects the defined source population and can reliably present all patients presenting with locally advanced pancreatic cancer in a defined population and over a defined time.

Thus, the aim of this study was to investigate the outcome for all patients diagnosed with locally advanced pancreatic cancer. The proportion of patients referred from other regions was expected to be higher for younger and fitter patients. Therefore, and to minimise risk of patient selection bias, this study sought to reflect the source population of the Stockholm area. The hypothesis was that patients recommended neoadjuvant chemotherapy for locally advanced pancreatic cancer had a longer overall survival compared to patients recommended palliative chemotherapy.

## Patients and Methods

### Study Design and Ethical Approval

This study was an observational cohort study. The study was performed in accordance with the Strengthening the Reporting of Observational Studies in Epidemiology guidelines^15^ and was approved by the Regional Ethics Committee in Stockholm, Sweden (reference number 2019-03345).

### Study Population

**Fig. 1:**
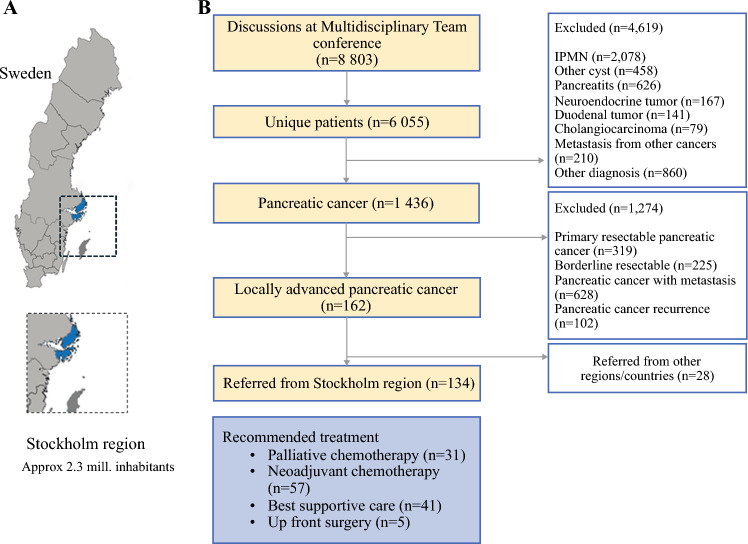
**A** Map of Sweden with the population-derived cohort defined from the Stockholm region. **B** Flow chart of patient distribution

A population-defined cohort within a defined Swedish population (approximately 2.3 million inhabitants as of November 2017) was obtained.^16^ The sampled population was all patients discussed at the pancreatic MDT between November 2017 and December 2021 at the Hepato-Pancreato-Biliary Unit at the Karolinska University Hospital. Data were prospectively collected via electronic health records. Inclusion criteria were age ≥18 years, residence in Stockholm region (Fig. 1A) and a diagnosis (by contrast-enhanced computer tomography with or without histopathology) of the National Comprehensive Canver Network (NCCN) classification criteria for locally advanced pancreatic cancer (Table S1).^3^ All patients recommended neoadjuvant chemotherapy were investigated with a four-phase computed tomography of thorax and abdomen and for cases where metastatic disease could not be ruled out, an additional liver-specific magnetic resonance imaging (MRI) was performed. No routine use of positron emission tomography (PET) was used in the study period.

During the last decades, all regions in Sweden have launched a standardised national routine which demands that all patients with a suspected pancreatic cancer must be referred to a pancreatic centre for discussion at an MDT. This practice has led to better knowledge on the disease prevalence and a more structured treatment routine in that no patient will receive treatment for a diagnosed pancreatic cancer without a discussion at an MDT. This routine makes Sweden an ideal setting to study outcomes representing the whole source population. Since 2017, locally advanced pancreatic cancer has been centralised in Sweden and Karolinska University Hospital in Stockholm is one of two national referral centres.

### Data Variables and Definitions

All data were collected from the electronical medical charts. The data were prospectively recorded in the medical charts.

### Exposure and Outcome

The exposure was recommended treatment allocation at the MDT and stratified in ‘palliative chemotherapy’, ‘neoadjuvant chemotherapy’, ‘best supportive care’ or ‘upfront surgery’.

Clinical tumour staging was performed by dedicated board-certified pancreatic radiologists. All patients with a suspected pancreatic cancer were discussed at an MDT and the tumour was staged. All patients fulfilling the inclusion criteria were considered for neoadjuvant chemotherapy, palliative chemotherapy, best supportive care or in some cases upfront surgery. The neoadjuvant chemotherapy regimens used in this cohort follow the recommendations in the NCCN guidelines.^3^ Re-evaluation of treatment effect was done routinely after four or six cycles of neoadjuvant chemotherapy. The re-staging protocol was based on the Response Evaluation Criteria in Solid Tumours (RECIST) criteria.^17^ Our palliative chemotherapy treatment regimens routines in un-resectable pancreatic cancer follow international guidelines and have been reported previously.^18^ Best supportive care treatment was defined as symptom-based treatment only (i.e. no medical oncological treatment). Treatment allocation was done at the multidisciplinary conference or in few cases at the following meeting with the patient in the outpatient clinic. Treatment allocation was based on a combination of patient characteristics, including comorbidity, age and performance status.

The outcomes for comparison were overall survival, 6-months, 1-year and 2-year survival. Survival time was defined as date of diagnosis to date of death (of any cause).

### Baseline Characteristics

Data were collected on age, body mass index (BMI), sex, American Society of Anaesthesiologists physical status (ASA-PS)^19^ classification, Eastern Cooperative Oncology Group (ECOG) performance status,^20^ Charlson comorbidity index, serum CA 19-9, tumour location and tumour size.

### Statistical Analysis

All analyses were performed using Stata (Stata-Corp. 2017. Stata Statistical Software: Release 17. College Station, TX: Stata Corp LP).

Descriptive statistics for categorical variables are presented with counts and percentages (%), and continuous variables were presented with median and interquartile ranges (IQRs). Categorical variables were analysed using the chi-squared test.

Survival distribution was calculated using the Kaplan–Meier method using the log-rank test for testing for statistical significance. Analysis of relative risk measures was performed using multivariable logistic regression, presented with odds ratios (OR) and 95% confidence intervals (95% CI). Cox proportional hazards analysis was done for time-to-event, presented with hazard ratios (HR) with 95% CI. Variables included in the multivariable adjustment models were decided a priori and were age, sex, ASA, ECOG and Charlson comorbidity index. All statistical tests were two-tailed and *p* values < 0.050 were considered statistically significant.

## Results

### Patient Characteristics

**Fig. 2:**
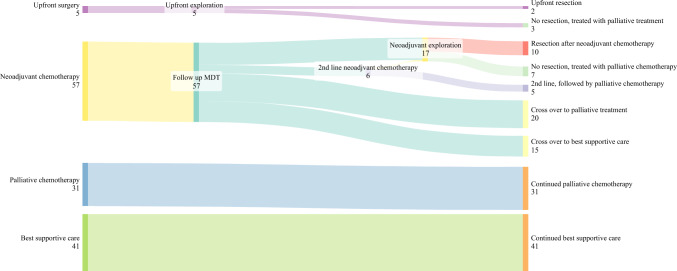
Sankey diagram showing the flow of patients based on treatment allocation

In total, 8806 events were discussed at a pancreatic MDT during the study period, of which some patients were discussed several times (Fig. 1). Hence, 6055 patients were unique and 1436 (24%) had pancreatic cancer. The number of patients fulfilling the NCCN criteria for locally advanced pancreatic cancer were 162 (11.3%), of which 134 were from the Stockholm health care region and hence included. Baseline characteristics of the included patients are described in Table 1. The response to treatment for patients in different treatment allocations is presented in Fig. 2. Patients in this cohort recommended neoadjuvant chemotherapy were on average younger and a had a better performance status than patients recommended palliative chemotherapy.

**Table 1:** Background characteristics for patients with locally advanced pancreatic cancer stratified according to treatment allocation at multidisciplinary tumour board

	Palliative chemotherapy	Neoadjuvant chemotherapy	Best supportive care	Upfront surgery	Overall
No. of patients	31 (23.1)	57 (42.5)	41 (30.6)	5 (3.7)	134 (100)
Age, years	71.3 [67.1–78.1]	66.8 [60.8–73.3]	82.8 [79.9–86.5]	71.5 [66.8–75.6]	73.4 [66.1–80.0]
BMI, kg/m^2^	22.2 [19.9–24.3]	25.0 [21.6–27.7]	22.3 [20.4–24.8]	24.6 [24.0–27.1]	23.1 [21.2–26.0]
Sex, women	20 (64.5)	26 (45.6)	23 (56.1)	2 (40.0)	71 (53.0)
ASA score					
1	2 (6.45)	18 (31.58)	0 (0)	1 (20.0)	21 (15.67)
2	14 (45.16)	33 (57.89)	19 (46.34)	3 (60.0)	69 (51.49)
3	15 (48.39)	5 (8.77)	21 (51.22)	1 (20.0)	42 (31.34)
4	0 (0)	1 (1.57)	1 (2.44)	0 (0)	2 (1.49)
ECOG score					
0	13 (41.94)	49 (85.96)	9 (21.95)	5 (100)	76 (56.72)
1	11 (35.48)	6 (10.53)	10 (24.39)	0 (0)	27 (20.15)
2	6 (19.35)	1 (1.75)	13 (31.71)	0 (0)	20 (14.93)
3	1 (3.23)	1 (1.75)	7 (17.07)	0 (0)	9 (6.72)
4	0 (0)	0 (0)	2 (4.88)	0 (0)	2 (1.49)
Charlson comorbidity index	5 [4–6]	5 [4, 5]	7 [6, 7]	5 [4, 5]	5 [4–7]
CA19-9 (kU/l)	142 [69–455]	445 [74–1180]	446 [91–1670]	109 [104–765]	252 [76–1060]
Tumour location					
Caput	17 (54.8)	44 (77.2)	23 (56.1)	4 (80.0)	88 (65.7)
Corpus	7 (22.6)	9 (15.8)	14 (34.2)	1 (20.0)	31 (23.1)
Cauda	7 (22.6)	4 (7.0)	4 (9.8)	0 (0)	15 (11.2)
Tumour size (mm)	39 [30–55]	39 [30–50]	40 [30–45]	34 [22–40]	40 [30–50]

Values for continuous data are median [IQR] and count (%) for categorical data

*IQR* interquartile range, *BMI* body mass index, *ASA* American Society of Anaesthesiologists’ classification system, *ECOG* Eastern Cooperative Oncology Group

### Outcome Analysis

**Fig. 3:**
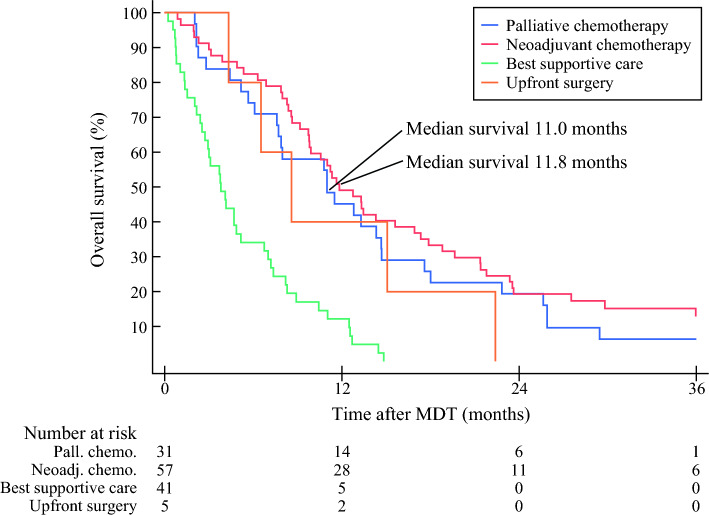
Kaplan–Meier survival analysis of mortality in all patients. The curves were statistically compared with log-rank test; *p* = 0.226 for neoadjuvant compared to palliative chemotherapy, *p* < 0.001 for best supportive care compared to palliative chemotherapy, *p* < 0.001 for upfront surgery compared with palliative chemotherapy

There was no statistically significant difference in any of the survival analyses for patients recommended neoadjuvant chemotherapy when compared to palliative chemotherapy (Table 2 and Fig. 3). The survival distribution estimates in the Kaplan–Meier curve did not deviate from each other when comparing patients recommended neoadjuvant to palliative chemotherapy. The analysis found no statistical difference between these groups and the median overall survival for patients recommended neoadjuvant chemotherapy was 11.8 months compared with 11.0 months for patients recommended palliative chemotherapy (*p* = 0.266, log-rank test). In the analyses, both the crude and adjusted analyses, there was no statistically significant difference in overall mortality in the analysis with multivariable logistic analysis of odds ratio (OR 0.27, 95% CI 0.02–3.29, *p* = 0.306) and Cox proportional hazard analysis (HR 0.96, 95% CI 0.58–1.59, *p* = 0.865) between patients in the neoadjuvant compared with the palliative chemotherapy group (Table 3). The other control group, patients who had been recommended the best supportive care, had a statistically significant shorter survival, including median survival (3.8 months), 6-months, 1-year and 2-year mortality. The hazard ratio for mortality was also statistically significantly elevated compared with patients recommended palliative chemotherapy.

**Table 2:** Outcome variables for patients with locally advanced pancreatic cancer stratified according to treatment intention at multidisciplinary team conference

	Palliative chemotherapy	Neoadjuvant chemotherapy	Best supportive care	Upfront surgery	Overall
No. of patients (%)	31 (23.1)	57 (42.5)	41 (30.6)	5 (3.7)	134 (100)
Median survival (months)	11.0 [7.6–14.7]	11.8 [9.8–16.9]*	3.8 [2.5–5.2]^#^	8.6	8.6 [7.7–11.0]
6 months survival	23 (74.2)	47 (82.5)*	14 (34.15)	4 (80.0)	88 (65.67)
1-year survival	14 (45.2)	28 (45.1)*	5 (12.20)^#^	2 (40.0)	49 (36.6)
2-year survival	6 (19.4)	11 (19.3)*	0^#^	0	17 (12.7)

Values for continuous data are median [IQR] and count (%) for number of patients

*p value (probability value) comparing neoadjuvant to palliative chemotherapy was > 0.500 for all outcomes

^#^*p* value comparing best supportive care to palliative chemotherapy was < 0.003 for all outcomes

**Table 3:** Multivariable logistic regression and multivariable cox proportional hazard analysis for overall mortality stratified on decision at multidisciplinary team conference

	OR/HR (95% CI) unadjusted	*p* value	OR/HR (95% CI) adjusted*	*p* value
OR				
Palliative chemotherapy (*n* = 31)	Ref.	Ref.	Ref.	Ref.
Neoadjuvant chemotherapy (*n* = 57)	0.24 [0.03–2.03]	0.189	0.27 [0.02–3.29]	0.306
Best supportive care (*n* = 41)	1	–	1	–
HR				
Palliative chemotherapy (*n* = 31)	Ref	Ref	Ref	Ref
Neoadjuvant chemotherapy (*n* = 57)	0.75 [0.48–1.19]	0.228	0.96 [0.58–1.59]	0.865
Best supportive care (*n* = 41)	3.29 [1.00–5.45]	< 0.001	2.26 [1.25–4.06]	0.006

*ASA* American Society of Anaesthesiologists’ classification system, *ECOG* Eastern Cooperative Oncology Group performance status, *95% CI* 95 % confidence interval

*The following covariates were included in the adjusted model: age, sex, ASA, ECOG and Charlson comorbidity index

### Effect of Neoadjuvant Chemotherapy and Surgery

**Fig. 4:**
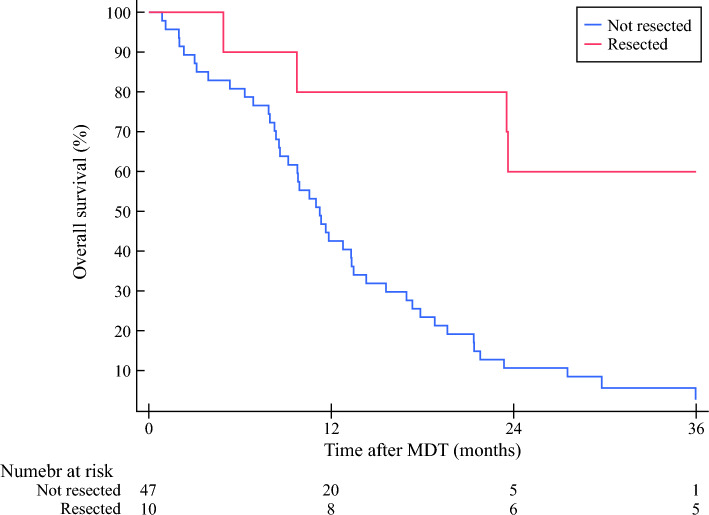
Kaplan–Meier survival analysis of mortality in neoadjuvant treated patients. Log-rank test, *p* < 0.001

Among the 57 patients recommended neoadjuvant chemotherapy, 26 patients (45%) were treated with mFOLFIRINOX, 13 (21%) were treated with gemcitabine–nab–paclitaxel, 11 patients (19%) were treated with regimens with combinations of chemotherapy with only one or two of components not fulfilling the two previously mentioned regimens. The remaining seven patients (12%) progressed or had a clinical deterioration in the beginning of the treatment and did not fulfil one full cycle of chemotherapy. The median number of cycles among patients treated with mFOLFIRINOX was 4 (IQR: 4–6) and among patients treated with Gemcitabine-Nab-Paclitaxel 3 (i.q.r 2-4) (*Table S2*). Out of all 57 patients, three were treated with radiotherapy in the neoadjuvant setting. Among all patients in the neoadjuvant group, 17 (29.8%) had a tumour response of the treatment that was deemed enough for exploration (Fig. 2). Of these 17 patients, 10 patients (17.5%) were resected, and 7 patients (12.3%) underwent surgical exploration without resection. In the survival analysis, neoadjuvant treated patients undergoing resection had a statistically significant longer survival compared to neoadjuvant treated patients not undergoing resection (Fig. 4). In the crude Cox proportional hazard analysis, the hazard ratio for overall mortality comparing resected to not resected patients was 0.15 (95% CI 0.05–0.45, *p* < 0.001). After adjusting for sex, age, Charlson comorbidity index, ASA and ECOG performance status the hazard ratio in a Cox regression model was 0.16 (95% CI 0.05–0.48, *p* = 0.001). The characteristics among the patients treated with neoadjuvant chemotherapy undergoing resection are presented in Table S3. Among the ten patients that were treated with neoadjuvant chemotherapy and underwent resection, six received adjuvant chemotherapy. The remaining four patients were considered not fit enough to receive adjuvant chemotherapy.

### Upfront Surgery

In the cohort, there were five patients recommended for upfront surgery despite fulfilling the NCCN’s criteria for locally advanced pancreatic cancer. Among these patients, three patients underwent surgery with only exploration (two due to carcinosis and one because of unresectable vessel involvement) and two patients underwent resection (Fig. 2).

## Discussion

In this cohort of locally advanced pancreatic cancer, from a population-defined region and studied in an intention-to-treat analysis, we investigated the survival according to the intended disease management. Of note, despite the finding that patients in this cohort recommended neoadjuvant chemotherapy had a lower median age and better performance status, there was no statistically significant difference in survival compared with patients recommended for palliative chemotherapy alone when compared with intention of treatment. This may imply both poor responders to neoadjuvant treatment but also some good responders in the palliative group, which may level out the overall difference between groups despite different intended treatment approaches.

One of the main strengths of this study is the study design, allowing for a complete insight into a population-based cohort based on a universal health care coverage treatment system of Sweden. Hence, there is minimal selection bias, and the results can be viewed as real-life outcomes of patients with locally advanced pancreatic cancer from a defined, non-selected population. As of today, in the current literature, there is a knowledge gap regarding the outcome for all patients with locally advanced pancreatic cancer. In a Cochrane review published a decade ago, the investigators concluded that there was a lack of studies on how patients with locally advanced pancreatic cancer ideally should be treated.^21^ After this review was published, new regimens have emerged and several large and well performed studies have been published in the field of neoadjuvant chemotherapy, surgery and survival in locally advanced pancreatic cancer showing promising results.^12,14,22–25^ Many of the last 10 years of surgical studies have, however, focussed on those patients undergoing resection and mainly the difference between the subgroup of neoadjuvant treated patients undergoing resection to patients not undergoing surgery. This comparison may restrict the external validity and introduce systematic bias, including immortal time bias.^26^ Based on deduction, the survival time should always be longer if a patient survived both neoadjuvant chemotherapy and hence was able to obtain a resection compared with patients who progressed during the neoadjuvant/induction chemotherapy time. Thus, counting survival from time of surgery, rather than time of diagnosis, may inflate the expected outcome based on selection. In this cohort, the immortal time bias was visualised in the survival figure of neoadjuvant treated patients, showing that all patients undergoing resection survived the first 6 months (Fig. 4). Statistical adjustments may address some of the different aspects in survival, but other biases including difference in tumour biology will remain unaccounted for. Many of the previous studies reporting on patients responding to chemotherapy and undergoing resection may also be at risk for selection bias regarding tolerability of preoperative treatment. It is therefore not well studied how a population-based cohort would respond to and tolerate the recommended treatments. In the current cohort, the tolerability and treatment distribution in the neoadjuvant chemotherapy group, shown in Table S2, fare well compared with a previous study reporting on borderline resectable and locally advanced pancreatic cancer in a population-based setting.^14^ This implies that the neoadjuvant cohort in this study were comparable with other centres’ population.

Perhaps even more importantly, a critical limitation when comparing resected with not resected patients stem from the fact that, at the time of diagnosis, both patient and physician are ignorant to whether a patient will respond to chemotherapy and undergo resection. At the time of diagnosis, knowledge of the outcome among all patients recommended a treatment regimen may be as relevant as the outcome among patients fulfilling and responding to a regimen. Informing patients about treatment options mandates knowledge of a patient’s wish and the field of pancreatic cancer, especially locally advanced pancreatic cancer, are lacking studies on patient needs and experience.^27,28^ It seems therefore equally important to investigate the outcome for all patients with locally advanced pancreatic cancer from an intention to treat analysis from the time of diagnosis as to investigate the outcome among patients undergoing surgery.

The external validity of single centre studies may be compromised by the unawareness whether the decisions reflect the routine in other centres. To address this and to place the results of this study into context we investigated this in several steps. First, the group of best supportive care was included and used as a control group, showing a significantly shorter overall survival than palliative and neoadjuvant chemotherapy. Second, the distribution of neoadjuvant chemotherapy regimen was similar to previous studies.^29^ Third, the resection rate in the neoadjuvant chemotherapy group was comparable with previous studies.^14,30^ These analyses indicate that the selection and treatment fulfilment in the decisions for patients recommended neoadjuvant chemotherapy was comparable with previous publications.

The findings of this study implies that current treatment for locally advanced pancreatic cancer may only benefit a selected few. The majority of patients recommended neoadjuvant chemotherapy, had no increased survival compared to palliative treatment alone. Hence, there is an urgent need in finding more effective drugs, define the responders and potentially increase the numbers that can actually undergo surgery for a curative attempt. As such, current guidelines are not specific enough at identifying the patients that will benefit from the neoadjuvant treatment. Better predictive models are needed^31^ and future guidelines need to address this.

This study has some limitations. The study analysed a historical cohort with the possible limitations inherent in the study design. However, the variables and outcome were prospectively recorded in the medical charts and therefore minimizes the risk of recall bias. There is also risk of selection bias. The analyses on resection frequency indicate, however, that compared with previous studies the patient selection was comparable with data reported in previous studies. Another limitation is the sample size and the possible risk of a type II statistical error, i.e. that despite not demonstrating a statistically significant difference in survival, there may still be one had the sample size been larger. Still, there are no previous publication with an equally comprehensive inclusion description including all patients with locally advanced pancreatic cancer regardless of treatment allocation. As such, the sample size should have less of a meaning per se, as the study cohort refers to an entire population at-risk over a specific time-period, and hence gives a comprehensive cross-sectional overview of what the expected outcomes may be for a patient presenting with locally advanced pancreatic cancer. The study sought to investigate the outcome in a defined population and presents all locally advanced pancreatic cancer patients in the Stockholm region during the study period. It represents the largest centre in northern Europe. Therefore, the external validity for other regions with a universal health care system should be high.

## Conclusions

In the current population-derived cohort of locally advanced pancreatic cancer investigated according to treatment intention at time of diagnosis, patients recommended for neoadjuvant chemotherapy at MDT had survival comparable with patients recommended for palliative chemotherapy. Patients responding to neoadjuvant chemotherapy and undergoing resection had a longer survival compared with nonresected neoadjuvant treated patients. Better selection and more effective drugs to treat patients with locally advanced pancreatic cancer is urgently needed to improve outcomes. Better tools to inform on decision before and during neoadjuvant therapy is needed.

## Supplementary Information

Below is the link to the electronic supplementary material.

Supplementary file1 (DOCX 20 KB)
