# Supplementary material for: Assessment of Outcomes by Intention-to-Treat Comparison for Locally Advanced Pancreatic Cancer: A Population-Derived Cohort Study
Source: Ann Surg Oncol. 2024 Oct 4;32(1):508–16. doi: 10.1245/s10434-024-16291-9 (PMC11659375; doi:10.1245/s10434-024-16291-9)
Supplement: Supplementary file 1 — Supplementary file1 (DOCX 20 KB) [file 10434_2024_16291_MOESM1_ESM.docx]

| **Table S1.** NCCN criteria for staging of pancreatic cancer (3) | | |
| --- | --- | --- |
| Resectability Status | Arterial | Venous |
| Resectable | No arterial tumor contact (celiac axis [CA], superior mesenteric artery  [SMA], or common hepatic artery [CHA]). | No tumor contact with the superior mesenteric vein (SMV) or portal vein (PV) or ≤180° contact without vein contour irregularity. |
| Borderline Resectable | Pancreatic head/uncinate process:  • Solid tumor contact with CHA without extension to CA or hepatic  artery bifurcation allowing for safe and complete resection and  reconstruction.  • Solid tumor contact with the SMA of ≤180°.  • Solid tumor contact with variant arterial anatomy (ex: accessory right hepatic artery, replaced right hepatic artery, replaced CHA, and the origin of replaced or accessory artery) and the presence and degree of tumor contact should be noted if present, as it may affect surgical planning.  Pancreatic body/tail:  • Solid tumor contact with the CA of ≤180°. | • Solid tumor contact with the SMV or PV of >180°, contact of ≤180° with contour irregularity of the vein or thrombosis of the vein but with suitable vessel proximal and distal to the site of involvement allowing for safe and complete resection and vein reconstruction.  • Solid tumor contact with the inferior vena cava (IVC). |
| Locally Advanced | Head/uncinate process:  • Solid tumor contact >180° with the SMA or CA.  Pancreatic body/tail:  • Solid tumor contact of >180° with the SMA or CA.  • Solid tumor contact with the CA and aortic involvement. | • Unreconstructible SMV/PV due to tumor involvement or  occlusion (can be due to tumor or bland thrombus). |

| **Table S2.** Treatment characteristics among patients treated with Neoadjuvant chemotherapy | | | |  |
| --- | --- | --- | --- | --- |
|  | mFOLFIRINOX | Gemcitabine-Nab-Paclitaxel | Other combinations |  |
| Number of patients | 26 | 13 | 11 |  |
| Median number of cycles | 4 [4-6] | 3 [2-4] | 3 [1-5] |  |
| Number of patients treated with radiotherapy | 2 (8) | 1 (8) | 0 (0) |  |
| Values for continuous data are median [IQR] and count (%) for number of patients | | | | |

| **Table S3.** Postoperative characteristics among patients treated with neoadjuvant chemotherapy that underwent resection | | | |  |
| --- | --- | --- | --- | --- |
|  | Pancreatoduodenectomy | Distal pancreatectomy | Total pancreatectomy |  |
| Total number of patients | 4 (100) | 2 (100) | 4 (100) |  |
| Arterial resection | 2 (50) | 2 (100) | 4 (100) |  |
| Venous resection | 1 (25) | 1 (50) | 3 (75) |  |
| Multivisceral resection | 0 | 1 (50) | 2 (50) |  |
| Clavien-Dindo >IIIA | 2 (50) | 2 (100) | 1 (25) |  |
| 90-day mortality | 0 | 0 | 0 |  |
| DGE grade B-C | 3 (75) | 0 | 2 (50) |  |
| PPH grade B-C | 0 | 0 | 0 |  |
| Bile leakage grade B-C | 0 | 0 | 1 (25) |  |
| POPF grade B-C | 1 (25) | 0 | - |  |
| Adjuvant chemotherapy | 2 (50) | 1 (50) | 3 (75) |  |
| Values for count (%) for number of patients. DGE; Delayed gastric emptying, PPH; Post pancreatectomy haemorrhage, POPF; postoperative pancreatic fistula | | | | |
